# Overloaded vertebral body: a unique radiographic phenomenon following multilevel anterior cervical discectomy and fusion

**DOI:** 10.1186/s13018-023-04365-5

**Published:** 2023-11-18

**Authors:** Shihao Chen, Kangkang Huang, Hao Liu, Tingkui Wu, Junbo He, Minghe Yao, Beiyu Wang

**Affiliations:** Department of Orthopedics, Orthopedic Research Institute, West China Hospital, Sichuan University, 37 Guoxue Lane, Chengdu, 610041 Sichuan China

**Keywords:** Overloaded vertebral body, Radiological outcomes, Anterior cervical discectomy and fusion, Three-level surgery

## Abstract

**Purpose:**

Because previous studies have not focused on postoperative cervical collapse, the purpose of the present study was to introduce the overloaded vertebral body (OVB) phenomenon following multilevel zero-profile anterior cervical discectomy and fusion (ACDF) as well as to investigate its effects on radiographic outcomes.

**Methods:**

We conducted a retrospective study involving patients who underwent ACDF. A total of 55 patients were included in the analysis, including 110 OVB and 110 non-OVB. The evaluated vertebral parameters included the vertebral cross-sectional area (CSA), wedge angle (WA), vertebral height [anterior (AH) and posterior (PH)] and anterior–posterior vertebral diameter [upper (UD) and lower (LD)].

**Results:**

The CSA and WA were significantly lower in the OVB group than in the non-OVB group at 3, 6, and 12 months after surgery as well as at the final follow-up (*p* < 0.01). The AH of the OVB group was significantly lower at 3, 6, and 12 months after surgery as well as at the final follow-up compared to 1 week after surgery (*p* < 0.01).

**Conclusions:**

OVB, a new phenomenon following multilevel ACDF, is defined as the cervical vertebral body located in the middle of the surgical segments in multilevel anterior cervical spine surgery. Statistical analysis of vertebral parameters, including CSA, WA, AH, PH, UD, and LD, showed that OVB occurs mainly at the anterior edge of the vertebra and that its largest radiographic manifestation is the loss of height at the anterior edge of the vertebra in the early postoperative period.

## Introduction

Aging is a serious worldwide problem, and as patients age, they develop cervical degenerative disc disease (CDDD) [1, 2]. The cervical spine is complex as it allows a great range of motion, and its main function is to support the weight of the head. However, CDDD causes compression of radiculopathy and/or myelopathy symptoms, which can cause neck discomfort, radiating upper extremity pain, numbness, and decreased muscle strength, reducing the patient's ability to perform daily activities and even leading to paralysis [3, 4]. Anterior cervical discectomy and fusion (ACDF) is an effective surgical option for the treatment of multilevel degenerative disc disease. It has been reported that multilevel ACDF with anterior mini-plates has some biomechanical advantages over conventional long fixation, such as reducing the occurrence of postoperative dysphagia and preserving more adjacent segment range of motion (ROM), resulting in a lower risk of adjacent segment degeneration (ASD) [5, 6].

Although multilevel ACDF is a common procedure, the potential complications associated with anterior surgery are not negligible, especially loss of segmental angle (SA) [7]. It has been widely accepted that cervical lordosis plays an important role in maintaining sagittal head and spinal balance [8]. Compromise on this lordotic curvature of the cervical spine, such as hypolordosis or kyphosis, is usually associated with neck pain, disability, and cervical disc degeneration [9]. In contrast, several retrospective studies have shown that a significant decrease in SA can be observed after multilevel ACDF [10, 11]. There are many speculative reasons for this occurrence, including osteoporosis, blood supply, damage to the endplate, and stress concentration [12, 13].

In the present study, we observed the uniqueness through the vertebral parameters and vertebral ratios after multilevel ACDF surgery with Zero-P fusion cage, which may explain the loss of cervical curvature. Due to the distinctive characteristics, we defined the cervical vertebra located in the middle of the surgical segments in multilevel zero-profile anterior cervical spine surgery as overloaded vertebra (OV), indicating that the upper and lower segments of OV are operated on. This study suggests that the primary changes occur in the vertebral body, so the main focus of this research is on overloaded vertebral body (OVB). To our knowledge, few studies have reported on postoperative vertebral conditions in multilevel ACDF. The purpose of the present study was to introduce the OVB phenomenon following multilevel ACDF as well as to investigate its effects on radiographic outcomes.

## Materials and methods

### Participants and procedure selection

**Fig. 1:**
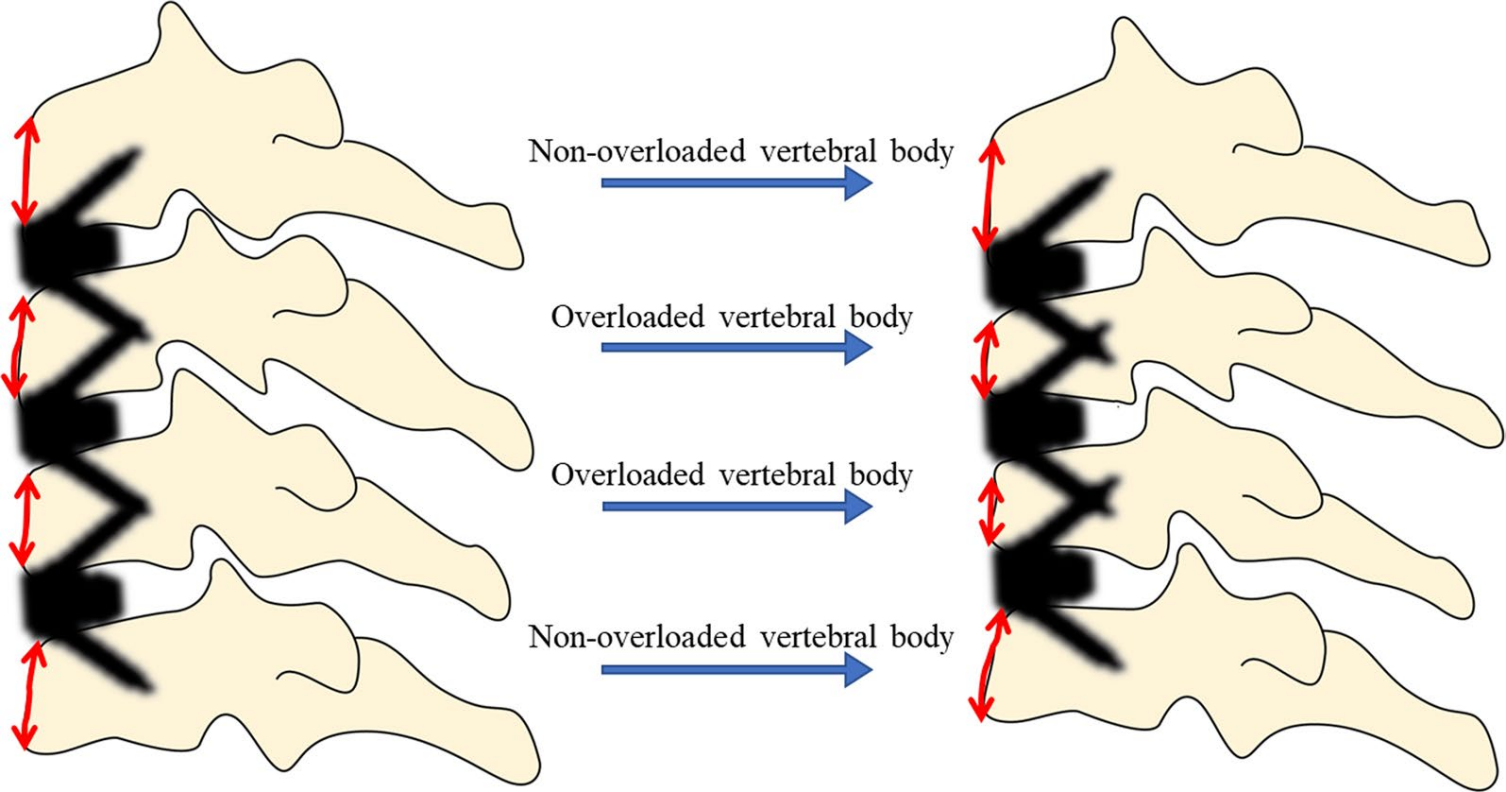
Schematic diagram of the overloaded vertebral body grouping pattern

We conducted a retrospective study involving patients with three-level CDDD who underwent ACDF with Zero-P or Zero-P VA implants (Synthes, Oberdorf, Switzerland) at our institution between March 2015 and June 2021. The vertebrae in the middle of the operated segment of the patients with three-level ACDF were considered the OVB group, and the upper and lower vertebrae of the OVB were considered the non-OVB group. A total of 55 patients were included in the analysis, including 110 OVB and 110 non-OVB (Fig. 1). The inclusion criteria were as follows: (1) diagnosis of spondylotic radiculopathy or myelopathy; (2) refractory to conservative treatment for at least 6 weeks; (3) confirmation of the lesion area by clinical symptoms and imaging (computed tomography, magnetic resonance imaging, and radiology); and (4) surgery at three levels between C3 and C7. The exclusion criteria were as follows: (1) previous cervical spine surgery; (2) presence of cervical stenosis, osteoporosis, tumor, or infection; (3) history of trauma or deformity; and (4) follow-up < 12 months. Ethical approval for this study was granted by the Ethics Committee of our institution. All participants provided informed consent for the analysis of their clinical data.

### Surgical technique

**Fig. 2:**
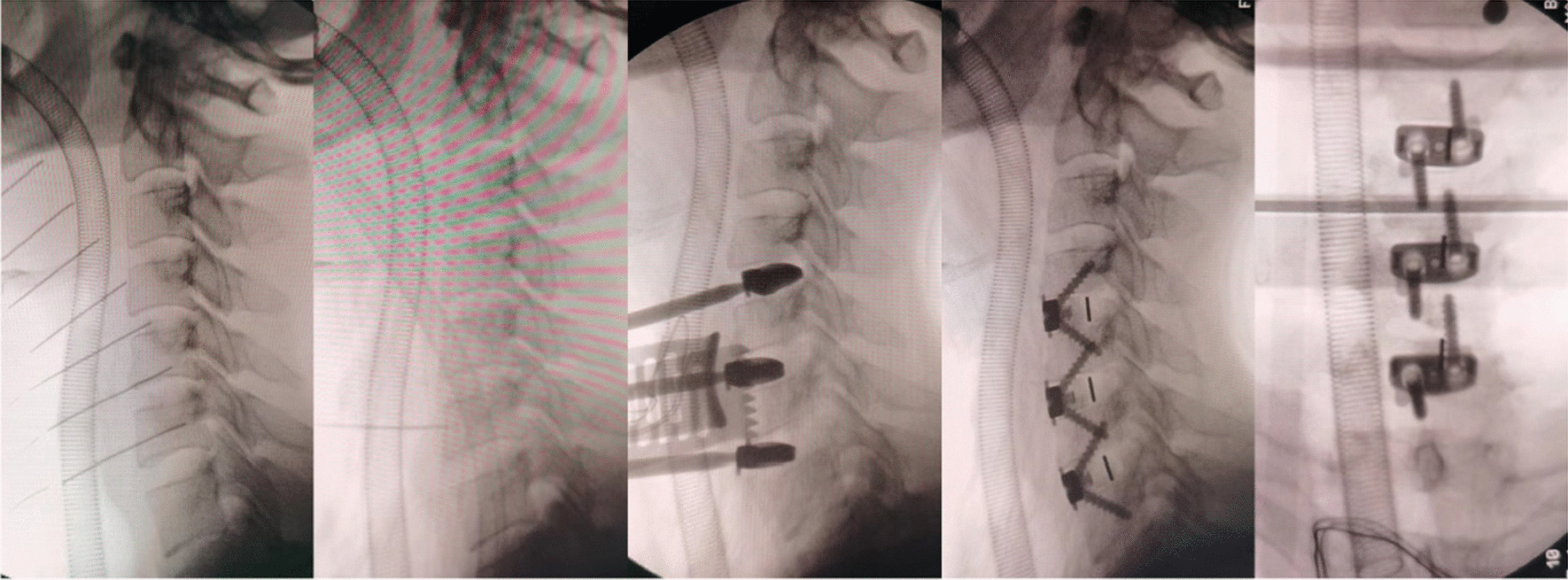
C-arm fluoroscopy image illustrating the surgical procedure, including confirmation of the precise implant placement

All anterior fusions were performed using the Smith-Robinson technique and a right-sided approach by the same surgeon. All patients underwent preoperative tracheoesophageal advancement training to prevent postoperative sputum and swallowing difficulties. After confirming and exposing the appropriate vertebra level, a Caspar retractor was used, and the disc material was removed. The endplate cartilage was scraped using a spatula or high-speed electric drill to prepare the endplate for bone grafting. The posterior longitudinal ligament, osteophytes, and other compressed elements were removed to ensure adequate dural and neural decompression. After measuring intervertebral height and width, an appropriate tricalcium phosphate-filled Zero-P or Zero-P VA implant was inserted with an implant scaffold/targeting device. C-arm fluoroscopy was performed to verify the correct position of the implant (Fig. 2). Finally, the incision was closed after insertion of the drainage tube.

### Data collection

The data were collected at 1 week, 3 months, 6 months, and 12 months postoperatively as well as at the final follow-up. Perioperative parameters, including the gender, age, BMI, operative segment, operative time and blood loss, were collected.

### Radiological evaluation

Cervical parameters were measured on lateral radiographs. All images were transferred to a picture archiving and communication system (PACS), and they were measured directly using the built-in tools of the PACS workstations. The following cervical parameters were evaluated: (1) vertebral cross-sectional area (CSA); (2) wedge angle (WA); (3) anterior vertebral height (AH); (4) posterior vertebral height (PH); (5) upper anterior–posterior vertebral diameter (UD); and (6) lower anterior–posterior vertebral diameter (LD).

**Fig. 3:**
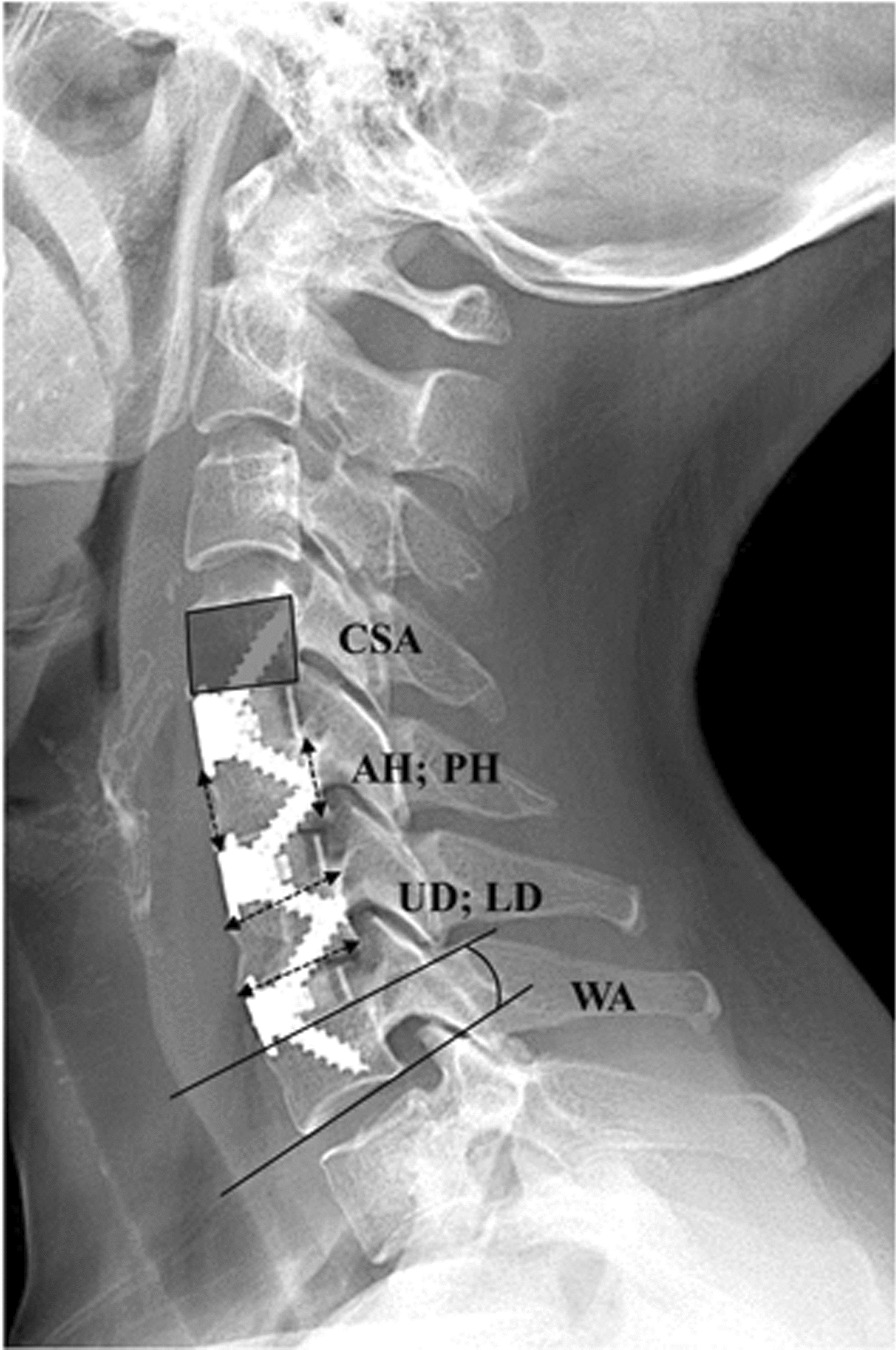
Schematic diagram of the parameters. X-rays show several parameters measured in this investigation. *CSA* Vertebral cross-sectional area, *AH* Anterior vertebral height, *PH* Posterior vertebral height, *UD* Upper anterior–posterior vertebral diameter, *LD* Lower anterior–posterior vertebral diameter, *WA* Wedge angle

CSA and WA were defined as the areas enclosed by the upper and lower endplates as well as the anterior and posterior edge of the vertebra and the angle of the tangent line connecting the upper and lower endplates of the vertebra, respectively. For WA, positive values represent lordosis, whereas negative values indicate kyphosis. Vertebral height was defined as the linear distance from the upper endplate of the vertebra to the lower endplate of the vertebra, and it was divided into AH and PH. Anterior–posterior vertebral diameter was defined as the linear distance from the anterior edge of the vertebra to the posterior edge of the vertebra, and it was divided into UD and LD. By measuring the height of the anterior edge of the vertebra in the OVB group at the final follow-up and 1 week postoperatively, the degree of vertebral height loss was classified as < 1/5, 1/5–1/3, or > 1/3 [14]. The wedge ratio (WR) was calculated using the following formula: WR = AH/PH × 100%. The extent of vertebral deformity (V-deformity) was presented as WR and graded from 0 to 3 according the grading presented by Genant et al. [15]: grade 0, normal (WR, ≥ 80%); grade 1, mildly deformed (WR, 75% – < 80%); grade 2, moderately deformed (WR, 60 to  < 75%); and grade 3, severely deformed (WR, < 60%) (Fig. 3).

### Statistical analysis

All statistical analyses were performed using SPSS (version 24.0, SPSS, Chicago, IL, USA). Continuous variables are presented as the mean ± standard deviation (SD), and categorical variables are presented as the number of cases. A paired t test was used to compare the cervical parameters after surgery. Student’s t test or the Mann‒Whitney U test was used to compare continuous variables depending on the normality of the data. A chi-square test or Fisher’s exact test was used to analyze categorical data. Statistical significance was defined as *p* < 0.01.

## Results

### Demographic and surgical data

A total of 55 patients were included in the analysis according to the inclusion and exclusion criteria, including 110 vertebrae in the OVB group and 110 vertebrae in the non-OVB group. The mean age of the 55 patients was 58.87 years ± 11.00 years, and the mean follow-up time was 26.78 months ± 16.76 months. The study details are shown in Table 1.

**Table 1:** Summary of the patient demographic data

	ACDF
No	55
Gender (male/female), n	27/28
Age, year	58.87 ± 11.00
BMI	24.33 ± 3.76
Levels (C3-6/C4-5), n	16/39
Operation time, min	160.11 ± 24.62
Blood loss, mL	66.55 ± 15.54
FU, months	26.78 ± 16.76

*ACDF* Anterior cervical discectomy and fusion, *BMI* Body mass index, *FU* Follow-up

### Radiological outcomes

**Fig. 4:**
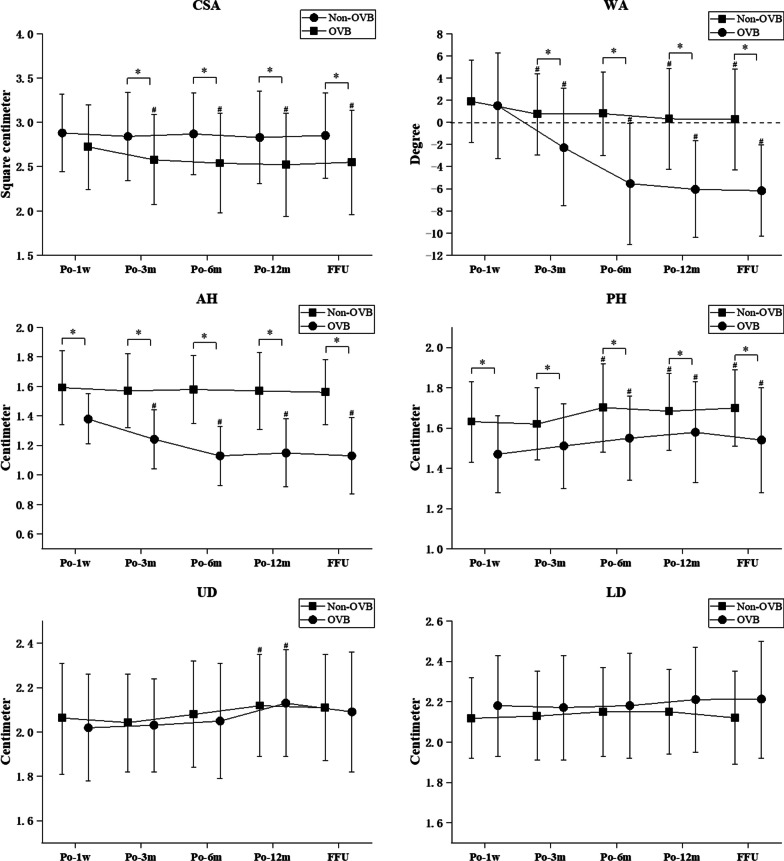
Radiological analysis of vertebral cross-sectional area, wedge angle, anterior vertebral height, posterior vertebral height, upper anterior–posterior vertebral diameter, and lower anterior–posterior vertebral diameter in patients. *OVB* Overloaded vertebral body, *CSA* Vertebral cross-sectional area, *WA* Wedge angle, *AH* Anterior vertebral height, *PH* Posterior vertebral height, *UD* Upper anterior–posterior vertebral diameter, *LD* Lower anterior–posterior vertebral diameter. *Significance on parameters between Non-OVB and OVB (*p* < 0.01). # Significance on parameters between Po-1w (*p* < 0.01)

Compared to the non-OVB group, the CSA was significantly lower in the OVB group at 3, 6, and 12 months after surgery as well as at the final follow-up (*p* < 0.01). In the OVB group, the CSA was significantly lower at 3, 6, and 12 months after surgery as well as at the final follow-up compared to 1 week after surgery (*p* < 0.01). The WA was significantly lower in the OVB group than in the non-OVB group at 3, 6, and 12 months after surgery as well as at the final follow-up (*p* < 0.01). The WA was significantly lower in the non-OVB and OVB groups at 3 months and 12 months after surgery as well as at the final follow-up compared to at 1 week after surgery (*p* < 0.01). Compared to 1 week after surgery, the WA was significantly lower in the OVB group at 6 months after surgery (*p* < 0.01). In addition, there was a significant reduction in the CSA and WA in the OVB group compared to the non-OVB group at the final follow-up (Table 2 and Fig. 4).

**Table 2:** Summary of the patient radiological analysis of vertebral cross-sectional area and wedge angle

	Non-OVB	OVB	*p* value
*CSA*			
Po-1w	2.88 ± 0.43	2.72 ± 0.46	0.012^a^
Po-3 m	2.84 ± 0.49	2.58 ± 0.50^#^	**0.000**^a^
Po-6 m	2.87 ± 0.46	2.54 ± 0.55^#^	**0.000**^a^
Po-12 m	2.83 ± 0.51	2.52 ± 0.59^#^	**0.000**^a^
FFU	2.85 ± 0.48	2.55 ± 0.60^#^	**0.000**^a^
*WA*			
Po-1w	1.90 ± 3.75	1.47 ± 4.63	0.444^a^
Po-3 m	0.73 ± 3.61^#^	− 2.23 ± 5.49^#^	**0.000**^a^
Po-6 m	0.95 ± 4.03	− 5.54 ± 5.55^#^	**0.000**^a^
Po-12 m	0.31 ± 4.51^#^	− 6.04 ± 4.41^#^	**0.000**^a^
FFU	0.27 ± 4.62^#^	− 6.15 ± 4.18^#^	**0.000**^a^

Bold values indicate statistically different

*OVB* Overloaded vertebral body, *CSA* Vertebral cross-sectional area, *WA* Wedge angle

^a^independent-samples T Test for the Non-OVB group and OVB group

^#^Significance on parameters between Po-1w (*p* < 0.01)

Compared to the non-OVB group, the AH and PH were significantly decreased at 1 week, 3 months, 6 months, and 12 months after surgery as well as at the final follow-up in the OVB group (*p* < 0.01). Compared to the immediate postoperative period (1 week after surgery), a decrease in AH was observed at all subsequent follow-ups (3 months, 6 months, and 12 months after surgery as well as at the final follow-up) in the OVB group (*p* < 0.01). Compared to 1 week after surgery, the PH was significantly higher at 6 months and 12 months after surgery as well as at the final follow-up in the OVB and non-OVB groups (*p* < 0.01). In addition, there was a statistically significant increase in the UD and LD only at 12 months after surgery compared to 1 week after surgery in the OVB and non-OVB groups (Tables 3 and 4, Fig. 4).

**Table 3:** Summary of the patient radiological analysis of anterior and posterior vertebral heights

	Non-OVB	OVB	*p* value
*AH*			
Po-1w	1.59 ± 0.24	1.38 ± 0.18	**0.000**^a^
Po-3 m	1.57 ± 0.25	1.24 ± 0.21^#^	**0.000**^a^
Po-6 m	1.58 ± 0.22	1.13 ± 0.22^#^	**0.000**^a^
Po-12 m	1.57 ± 0.26	1.15 ± 0.23^#^	**0.000**^a^
FFU	1.56 ± 0.21	1.13 ± 0.27^#^	**0.000**^a^
*PH*			
Po-1w	1.63 ± 0.20	1.47 ± 0.19	**0.000**^a^
Po-3 m	1.62 ± 0.18	1.51 ± 0.21	**0.000**^a^
Po-6 m	1.70 ± 0.23^#^	1.55 ± 0.21^#^	**0.000**^a^
Po-12 m	1.68 ± 0.19^#^	1.58 ± 0.26^#^	**0.000**^a^
FFU	1.70 ± 0.19^#^	1.54 ± 0.26^#^	**0.000**^a^

Bold values indicate statistically different

*OVB* Overloaded vertebral body, *AH* Anterior vertebral height, *PH* Posterior vertebral height

^a^independent-samples T Test for the Non-OVB group and OVB group

^#^Significance on parameters between Po-1w (*p* < 0.01)

**Table 4:** Summary of the patient radiological analysis of the upper and lower anterior–posterior vertebral diameters

	Non-OVB	OVB	*p* value
*UD*			
Po-1w	2.06 ± 0.26	2.02 ± 0.23	0.250^a^
Po-3 m	2.04 ± 0.22	2.03 ± 0.20	0.565^a^
Po-6 m	2.08 ± 0.23	2.05 ± 0.27	0.451^a^
Po-12 m	2.12 ± 0.22^#^	2.13 ± 0.25^#^	0.778^a^
FFU	2.11 ± 0.24	2.08 ± 0.27	0.376^a^
*LD*			
Po-1w	2.12 ± 0.20	2.18 ± 0.24	0.075^a^
Po-3 m	2.13 ± 0.23	2.17 ± 0.26	0.230^a^
Po-6 m	2.15 ± 0.22	2.18 ± 0.26	0.364^a^
Po-12 m	2.15 ± 0.21	2.21 ± 0.25	0.074^a^
FFU	2.12 ± 0.23	2.21 ± 0.29	0.011^a^

Bold values indicate statistically different

*OVB* Overloaded vertebral body, *UD* Upper anterior–posterior vertebral diameter, *LD* Lower anterior–posterior vertebral diameter

^a^independent-samples T Test for the Non-OVB group and OVB group

^#^Significance on parameters between Po-1w (*p* < 0.01)

According to vertebral height loss, there were 93 cases (84.5%) with < 1/5 height loss, 15 cases (13.6%) with 1/5–1/3 height loss, and 2 case (1.8%) with > 1/3 height loss at 3 months after surgery, while there were 68 cases (61.8%) with < 1/5 height loss, 32 cases (29.1%) with 1/5–1/3 height loss, and 10 cases (9.1%) with a height loss > 1/3 at the final follow-up. The number of vertebrae with a severe degree of height loss at the final follow-up was higher than that at 1 week after surgery. Similar results were obtained for the V-deformity as follows: there were 96 cases (87.3%) with grade 0, 11 cases (10.0%) with grade 1, 3 case (2.7%) with grade 2, and 0 cases (0.0%) with grade 3 at 1 week after surgery; and there were 26 cases (23.6%) with grade 0, 21 cases (19.1%) with grade 1, 51 cases (46.4%) with grade 2, and 12 cases (10.9%) with grade 3 at the final follow-up (Table 5).

**Table 5:** Trend of overloaded vertebral body anterior vertebral height with follow-up time

	OVB AH (n = 110)				
	Po-1w	Po-3 m	Po-6 m	Po-12 m	FFU
*Height loss*					
< 1/5	–	93 (84.5%)	72 (65.5%)	71 (64.5%)	69 (62.7%)
1/5–1/3	–	15 (13.6%)	28 (25.5%)	31 (28.2%)	31 (28.2%)
> 1/3	–	2 (1.8%)	10 (9.1%)	8 (7.3%)	10 (9.1%)
*V-deformity*					
Grade 0	96 (87.3%)	69 (62.7%)	29 (26.4%)	26 (23.6%)	26 (23.6%)
Grade 1	11 (10.0%)	14 (12.7%)	18 (16.4%)	24 (21.8%)	21 (19.1%)
Grade 2	3 (2.7%)	22 (20.0%)	52 (47.3%)	49 (44.5%)	51 (46.4%)
Grade 3	0 (0.0%)	5 (4.5%)	11 (10.0%)	11 (10.0%)	12 (10.9%)

*OVB* Overloaded vertebral body, *AH* Anterior vertebral height

## Discussion

**Fig. 5:**
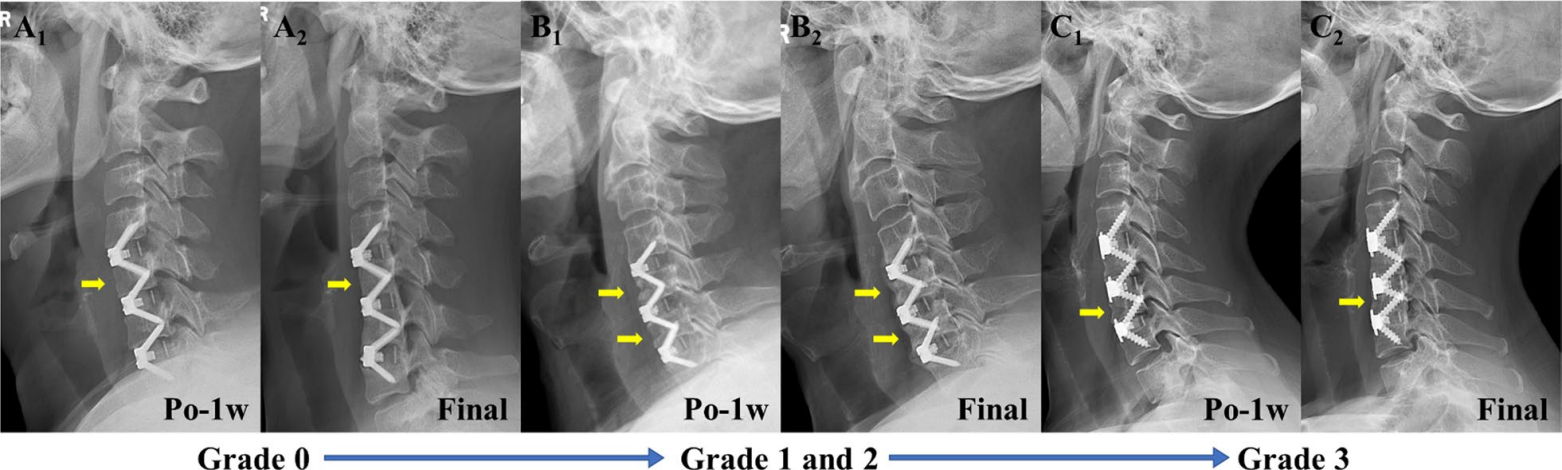
Installation according to the severity of vertebral deformity. Yellow arrows indicate vertebrae with significant morphological changes. **A** A middle-aged female patient with C4/5, C5/6, and C6/7 ACDF had all C4/5, C5/6, and C6/7 fused at the final follow-up. The AH of the superior OVB decreased significantly from 1.60 cm at 1 week postoperatively to 1.41 cm at the final follow-up, and there was significant bone resorption at the anterior edge. **B** A middle-aged male patient with C4/5, C5/6, and C6/7 ACDF. The AH of the superior OVB decreased significantly from 1.60 cm at 1 week postoperatively to 1.21 cm at the final follow-up; the AH of the inferior OVB decreased significantly from 1.46 cm at 1 week to 1.11 cm at the final follow-up. **C** A middle-aged female patient with C4/5, C5/6, and C6/7 ACDF had C5/6 and C6/7 fused at the final follow-up. The AH of the inferior OVB decreased significantly from 1.28 cm at 1 week postoperatively to 0.79 cm at the final follow-up

ACDF is a widely accepted surgical procedure for the treatment of multilevel CDDD [16–18]. Multilevel ACDF is effective in restoring interbody height and physiological curvature of the cervical spine as well as reestablishing stability of the cervical spine, resulting in a lower risk of structural failure and adjacent segment degeneration [19, 20]. With an aging population, the presence of cervical kyphosis, and the recognition of the importance of cervical sagittal alignment and maintaining sagittal balance, the consideration and performance of multilevel ACDF continues to improve. However, with the increasing utilization of multilevel ACDF surgery and the increase in follow-up, complications, such as ASD, dysphagia, subsidence, implant failure, and hoarseness, after the procedure have emerged [21]. Lin et al. [13] reported early postoperative middle cervical vertebral body collapse in 4 out of 27 cases after two-level ACDF, but they did not provide a specific definition of this occurrence nor provide a comprehensive parametric measurement and statistical analysis. In this study, we defined the cervical vertebral body located in the middle of the surgical segments in multilevel anterior cervical spine surgery as the OVB. By comparing radiographic findings between OVB and non-OVB, we identified a new phenomenon of OVB that may impact the prognosis after ACDF surgery using the Zero-P fusion cage (Fig. 5).

In the present study, three-level ACDF resulted in a significantly lower CSA and WA in the OVB group than in the non-OVB group at the postoperative follow-ups, which indicated that OVB does exist after multilevel ACDF and is mainly manifested by morphological changes in the vertebra. Moreover, the measurement of vertebral imaging parameters showed that the OVB was more likely to suffer with the reduction of vertebral height in multilevel ACDF. For osteoporotic vertebral endplate and cortical fractures, approximately 60–70% showed compression of the cervical anterior edge on imaging, and 11.4% of vertebral fractures had > 1/3 loss of vertebral height [22]. Studies have shown that the anterior vertebral wall is a biomechanically weak site [23]. For the AH, In the OVB group, the AH was significantly lower at 3 months, 6 months, and 12 months after surgery as well as at the final follow-up compared to 1 week after surgery, indicating a significant decrease in AH over time in OVB. In contrast, there were no statistically significant differences in the postoperative follow-up data for the other vertebrae, suggesting that the occurrence of this phenomenon in OVB is concentrated at the anterior edge of the vertebra with physiological weakness. In addition, combining the two classifications of vertebral ratios indicated that a large percentage of vertebral anterior edge reduction was present at the final follow-up in the early postoperative period.

The phenomenon of OVB investigated in the present study was different from the previously studied subsidence and collapse. Lu et al. [24] conducted a meta-analysis that included 626 patients, and they reported that multilevel ACDF with anterior mini-plates has a higher risk of postoperative subsidence than conventional long fixation. Noordhoek et al. [25] showed that the mean incidence of cage subsidence after ACDF surgery with Zero-P fusion cage is 21% (range 0 to 83%). Subsidence and collapse are similar in that they both result in loss of disc height, loss of segmental lordosis, and a decrement of neural foramen dimension after ACDF [26, 27]. Subsidence is mostly the sinking of a body with a higher modulus of elasticity (e.g., graft, cage, and spacer) into a body with a lower modulus of elasticity (e.g., vertebra), and collapse is primarily the compression of a body with a similar or lower modulus of elasticity (e.g., autologous or allograft bone) by a surrounding body with a similar or higher modulus of elasticity (e.g., vertebra), resulting in its own compression [28, 29]. These previous two studies concentrated more on the condition of the intervertebral space and involved fewer morphological changes in the vertebra, indicating that OVB is a new phenomenon that is fundamentally different from subsidence and collapse.

Previous studies have reported that increased intraoperative wear on the endplate may lead to higher rates of surgical failure or at least greater subsidence and collapse, especially in patients with osteoporosis and poor bone quality [28]. In contrast, the present findings indicated that it may be mainly due to bone remodeling after microdamage of the endplate bone caused by the surgical grinding of the endplate. This process may have been influenced by the altered biomechanical environment of the operated segment as the cervical spine was in anterior convexity and the anterior edge of the vertebra was under less force. According to Wolff's law, bone resorption occurred. Because the multiplate multiscrew model shares more load compared to the single-plate model, multiple screws will reduce the stress on the anterior plate [20]. The bone portion of the vertebra may have received destructive stresses due to screwing in multiple screws, which led to the collapse of the vertebra. Therefore, the postoperative vertebral morphological changes caused by the different surgical segments are different, and it is crucial to reduce the wear on the endplate and the use of screws during surgery. A biomechanical analysis of customized cages conforming to the endplate morphology may reduce the risk of stress shielding and the occurrence of subsidence [2, 30]. Preoperative customization of the vertebral cage by evaluating and measuring the morphology of the vertebra at different segments may be more consistent with human biomechanics and thus reduce the incidence of morphological change of OVB.

The present study had several limitations. First, the present study was a single-center retrospective study with a small sample size due to the small number of multilevel CDDD cases. Second, the present study did not analyze the clinical outcomes after three-segment ADCF. However, the present study was not designed to understand the efficacy of this procedure, which has been demonstrated in many other studies [31, 32]. Finally, the extended review period raises the possibility of varying vertebral changes among patients who underwent the intervention during this timeframe. Therefore, a multicenter prospective study should be performed to further confirm the present findings.

## Conclusions

The present study demonstrated that OVB exists and is defined as the cervical vertebral body located in the middle of the surgical segments in multilevel anterior cervical spine surgery. Statistical analysis of vertebral parameters, including CSA, WA, AH, PH, UD, and LD, showed that OVB occurs mainly at the anterior edge of the vertebra and that its largest radiographic manifestation is the loss of height at the anterior edge of the vertebra at 3, 6 months postoperatively.

## Data Availability

Datasets are available from the corresponding author on a reasonable request.

## Abbreviations

CDDD: Cervical degenerative disc disease
ACDF: Anterior cervical discectomy and fusion
ROM: Range of motion
ASD: Adjacent segment degeneration
SA: Segmental angle
OV: Overloaded vertebra
OVB: Overloaded vertebral body
PACS: Picture archiving and communication system
CSA: Vertebral cross-sectional area
WA: Wedge angle
AH: Anterior vertebral height
PH: Posterior vertebral height
UD: Upper anterior–posterior vertebral diameter
LD: Lower anterior–posterior vertebral diameter
WR: Wedge ratio
SD: Standard deviation
BMI: Body mass index
